# Supplementary material for: Adherens junction remodelling during mitotic rounding of pseudostratified epithelial cells
Source: EMBO Rep. 2020 Feb 7;21(4):e49700. doi: 10.15252/embr.201949700 (PMC7132200; doi:10.15252/embr.201949700)
Supplement: Supplementary file 1 — Expanded View Figures PDF [file EMBR-21-e49700-s001.pdf]

Expanded View Figures

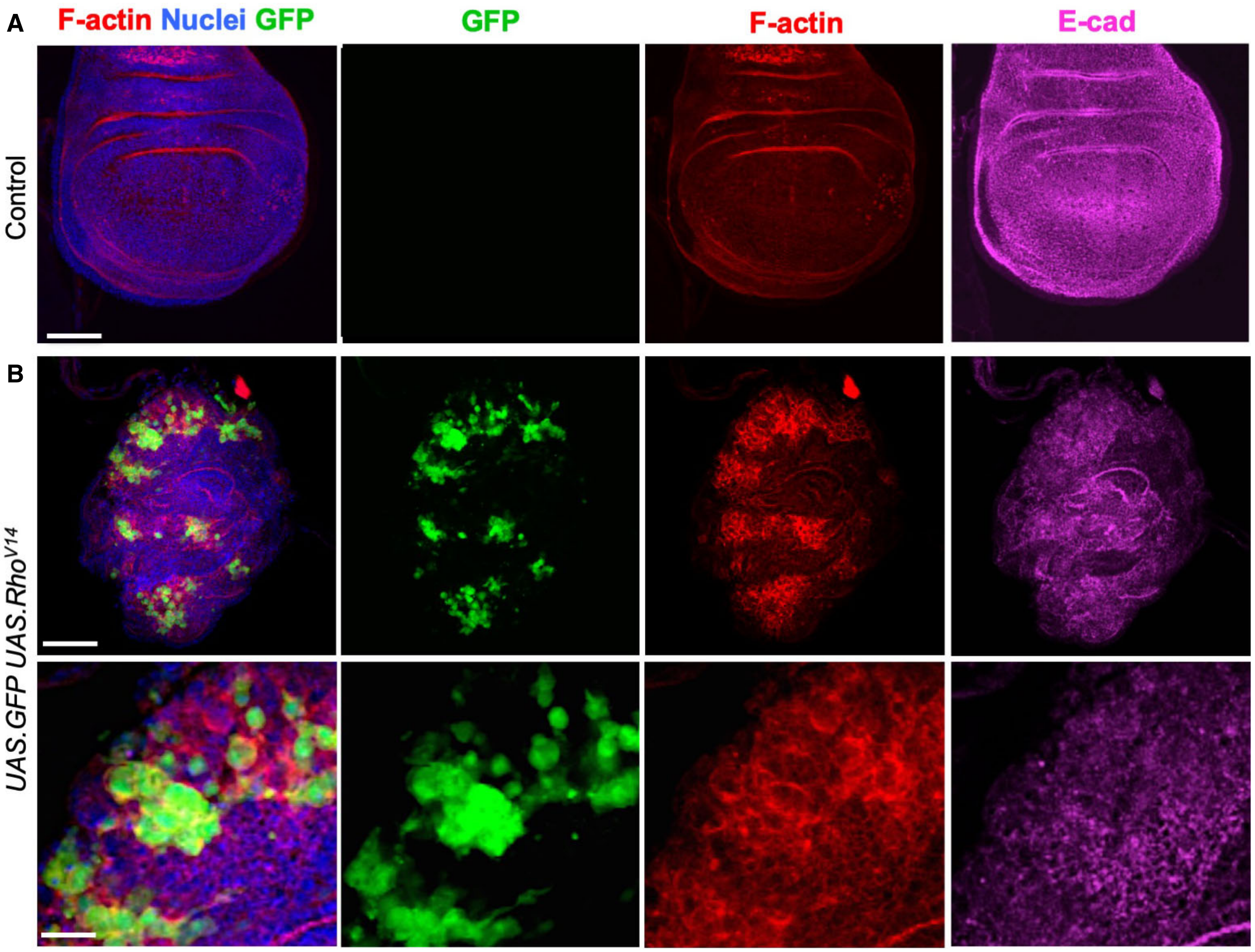

Figure EV1. Ectopic activation of Rho is sufficient to drive cell rounding and downregulate adherens junctions in the *Drosophila* wing disc.

A Control third instar wing imaginal disc stained for F-actin (phalloidin, red) and E-cadherin (magenta). Note absence of clones (GFP), there is no background signal for GFP. Scale bar ~20 µm. $n > 4$ independent biological replicates.

B Induction of clones expressing *UAS.GFP* and *UAS.RhoV14* causes dramatic increases in F-actin and co-incident decreases in E-cadherin, along with abnormal tissue morphology. High-magnification view (bottom) shows reduced E-cadherin and rounded shape of cells expressing RhoV14. Scale bars ~20 µm (low mag) ~10 µm (high mag). $n > 4$ independent biological replicates.

© 2020 The Authors

Figure EV2. RNAi of ECT2/Pbl leads to enlarged cells (cytokinesis defect) or extrusion of cells from the epithelium and subsequent apoptosis (spindle orientation defect) in the *Drosophila* wing disc.

A Third instar wing imaginal disc expressing *UAS.Pbl-RNAi* in the posterior compartment driven by the *hh.Gal4* line. Note enlarged cells (marked by F-actin, aPKC or Dlg staining). DAPI marks nuclei. Scale bars ~20 μm (low mag) ~5 μm (high mag). $n > 6$ independent biological replicates.

B Third instar wing imaginal disc expressing *UAS.Pbl-RNAi* in the entire wing pouch driven by the *MS1096.Gal4* line. Note many extruded cells (marked by F-actin, aPKC or Dlg staining). DAPI marks nuclei, including many pyknotic nuclei due to apoptosis. Scale bars ~20 μm (low mag) ~5 μm (high mag). $n > 7$ independent biological replicates.

C Control cross-section of wild-type third instar wing disc stained for aPKC, Dlg and nuclei (DAPI). Scale bar ~2 μm. $n > 4$ independent biological replicates.

D Cross-section of third instar wing imaginal disc expressing *UAS.Pbl-RNAi* in the entire wing pouch driven by the *MS1096.Gal4* line. Note extrusion of cells (revealed by staining for aPKC, Dlg) and many pyknotic nuclei (DAPI staining). Arrow shows residual polarised apical domains in cells that have been extruded. Scale bar ~2 μm. $n > 9$ independent biological replicates.

 © 2020 The Authors

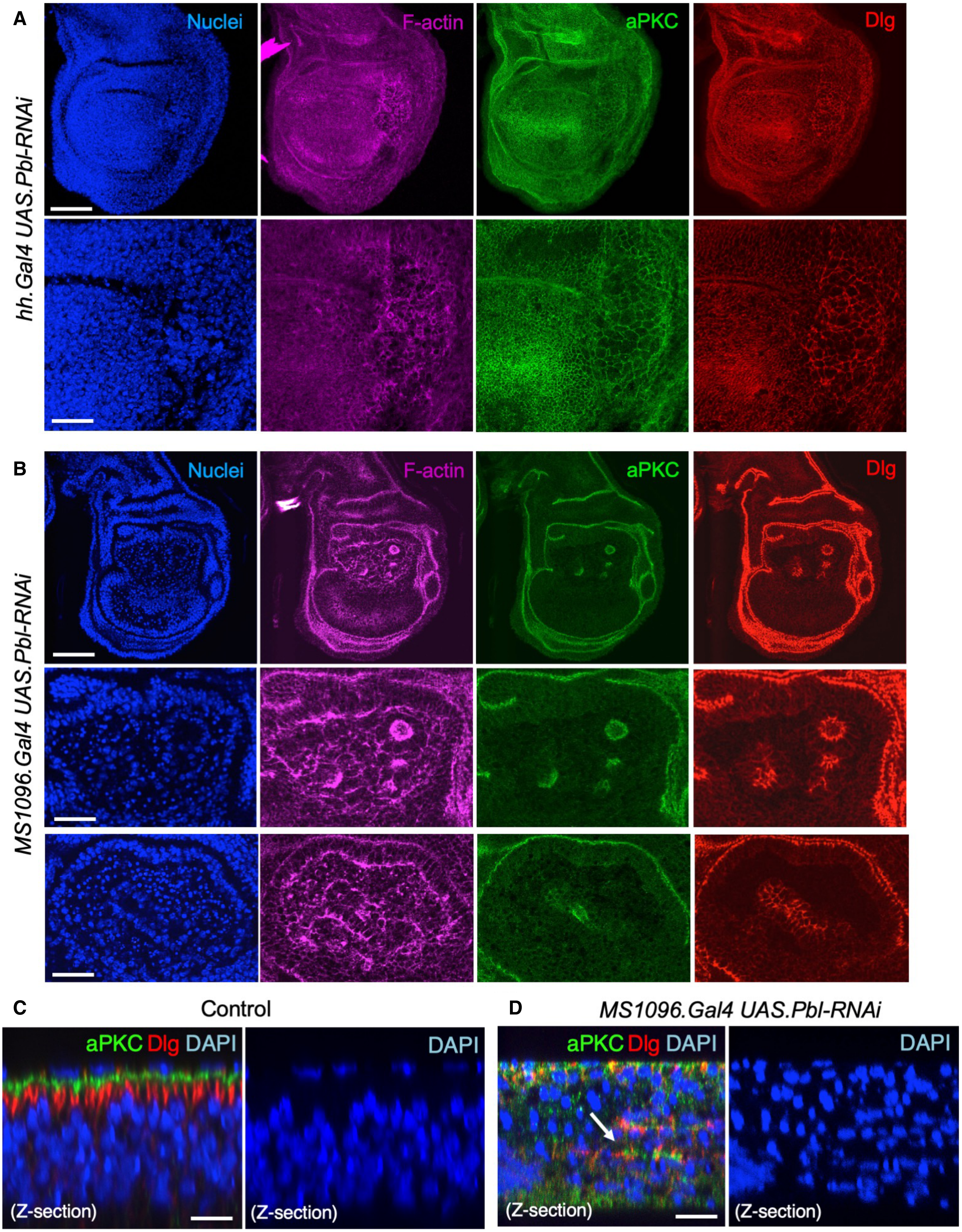
A
hh.Gal4 UAS.Pbl-RNAi
Nuclei
F-actin
aPKC
Dlg
B
MS1096.Gal4 UAS.Pbl-RNAi
Nuclei
F-actin
aPKC
Dlg
C
Control
aPKC Dlg DAPI
DAPI
(Z-section)
(Z-section)
D
MS1096.Gal4 UAS.Pbl-RNAi
aPKC Dlg DAPI
DAPI
(Z-section)
(Z-section)

Figure EV2.

Figure EV3. RNAi of both ECT2/Pbl and beta-Pix leads to reduced apical aPKC immunostaining in the *Drosophila* follicle cell epithelium.

A Cross-section of cuboidal follicular epithelium showing cytoplasmic localisation of overexpressed Cdc42 GEF beta-PIX tagged with HA. Scale bar ~3 µm. $n > 4$ independent biological replicates.

B Cross-section of cuboidal follicular epithelium showing nuclear and cytoplasmic localisation of overexpressed ECT2/Pbl tagged with HA. Scale bar ~3 µm. $n > 10$ independent biological replicates.

C Stage 7 egg chamber in which follicle cells have mutant clones for pix^{P1036}, marked by expression of GFP, shows extrusion of cells which retain abnormal aPKC and E-cad localisation. Scale bar ~6 µm. $n > 4$ independent biological replicates.

D Stage 7 egg chamber in which follicle cells have mutant clones for pix^{P1036} also expressing *UAS.Pbl-RNAi*, marked by expression of GFP, shows extrusion of cells featuring loss of aPKC plasma membrane localisation, along with abnormal E-cad localisation. Scale bar ~6 µm. $n > 3$ independent biological replicates.

E Stage 7 egg chamber in which follicle cells have mutant clones for pix^{P1036} also expressing *UAS.Pbl-RNAi*, marked by expression of GFP, shows extrusion of cells featuring mislocalisation of p-MyoII (p-MLC), along with abnormal E-cad localisation. Scale bar ~6 µm. $n > 3$ independent biological replicates.

F High-magnification view of mutant clones for pix^{P1036} also expressing *UAS.Pbl-RNAi*, marked by expression of GFP, shows extrusion of cells featuring mislocalisation of p-MyoII (p-MLC). Scale bar ~3 µm. $n > 3$ independent biological replicates.

G Wild-type stage 7 egg chamber in which follicle cells have normal aPKC and Dlg localisation. Scale bar ~6 µm. $n > 4$ independent biological replicates.

H *tj.Gal4*-driven *UAS.pix-RNAi* causes mild extrusion of cells with abnormal aPKC localisation (arrow). Scale bar ~6 µm. $n > 5$ independent biological replicates.

I *tj.Gal4*-driven *UAS.pbl-RNAi* causes enlarged cells with normal morphology and aPKC localisation. Scale bar ~6 µm. $n > 3$ independent biological replicates.

J *tj.Gal4*-driven *UAS.pix-RNAi* and *UAS.pbl-RNAi* causes loss of aPKC throughout the follicular epithelium, while Dlg spreads ectopically. Scale bar ~6 µm. $n > 9$ independent biological replicates.

© 2020 The Authors

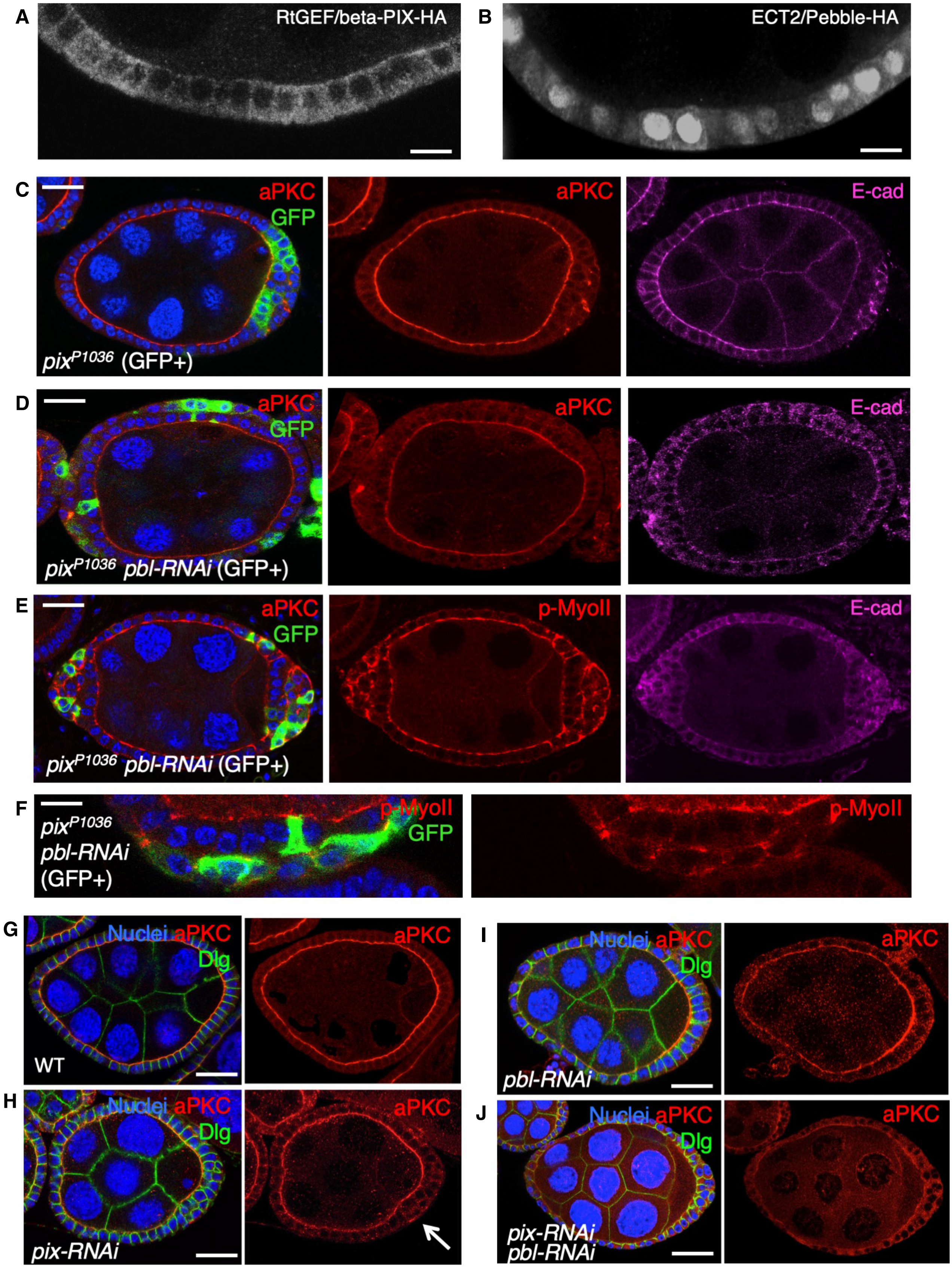
A
RtGEF/beta-PIX-HA
B
ECT2/Pebble-HA
C
aPKC
GFP
pix^{P1036} (GFP+)
aPKC
E-cad
D
aPKC
GFP
pix^{P1036} pbl-RNAi (GFP+)
aPKC
E-cad
E
aPKC
GFP
pix^{P1036} pbl-RNAi (GFP+)
p-MyoII
E-cad
F
pix^{P1036}
pbl-RNAi
(GFP+)
p-MyoII
GFP
p-MyoII
G
Nuclei aPKC
Dlg
WT
aPKC
H
Nuclei aPKC
Dlg
pix-RNAi
aPKC
I
Nuclei aPKC
Dlg
pbl-RNAi
aPKC
J
Nuclei aPKC
Dlg
pix-RNAi
pbl-RNAi
aPKC

Figure EV3.

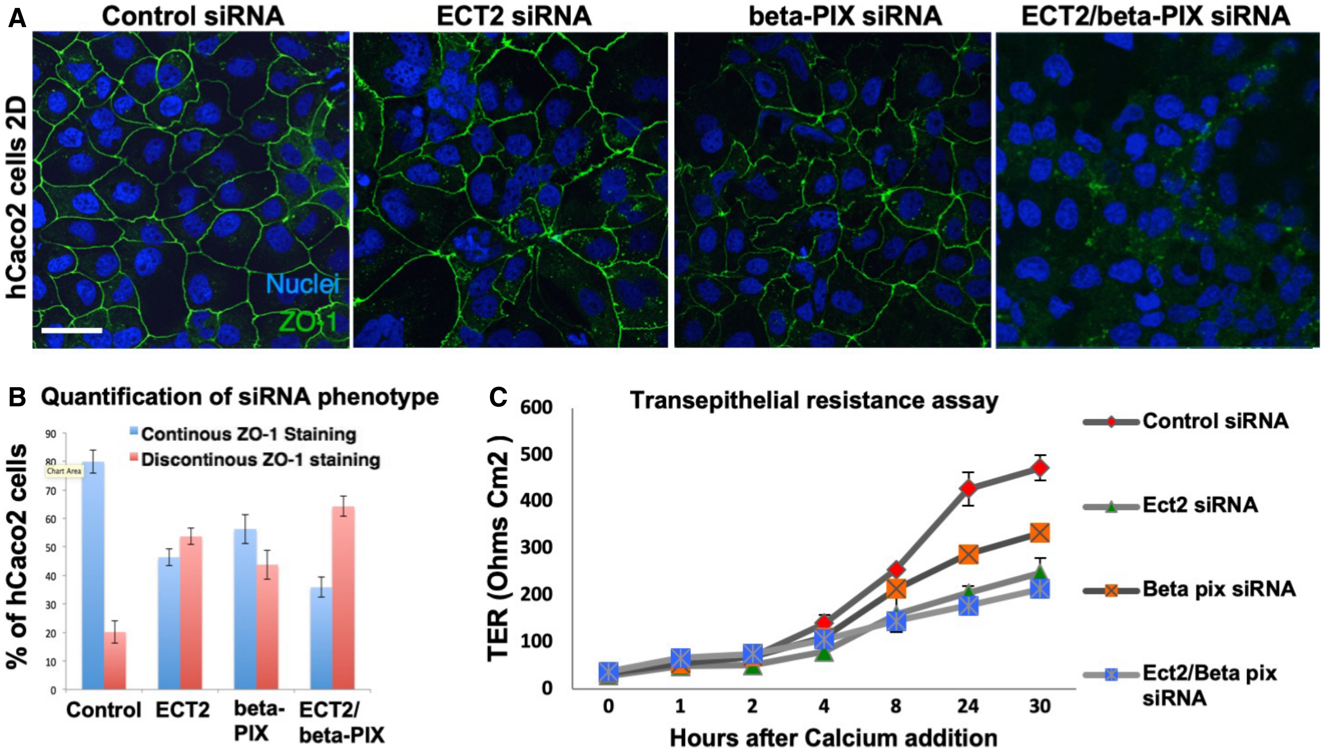

Figure EV4. RNAi of both ECT2/Pbl and beta-Pix leads to reduced apical ZO-1 immunostaining in human Caco2 cells.

A siRNA knockdown of ECT2 and beta-PIX individually does not affect the localisation of ZO-1, while double siRNA causes discontinuous ZO-1 localisation in most cells. Scale bar ~10 μm. $n > 3$ independent biological replicates.

B Quantification of (A). $n > 3$ independent biological replicates. Mean ± SEM shown.

C Transepithelial resistance assay for measuring tight junction function in each of the siRNA conditions in (A). $n > 3$ independent biological replicates. Mean ± SEM shown.

 © 2020 The Authors

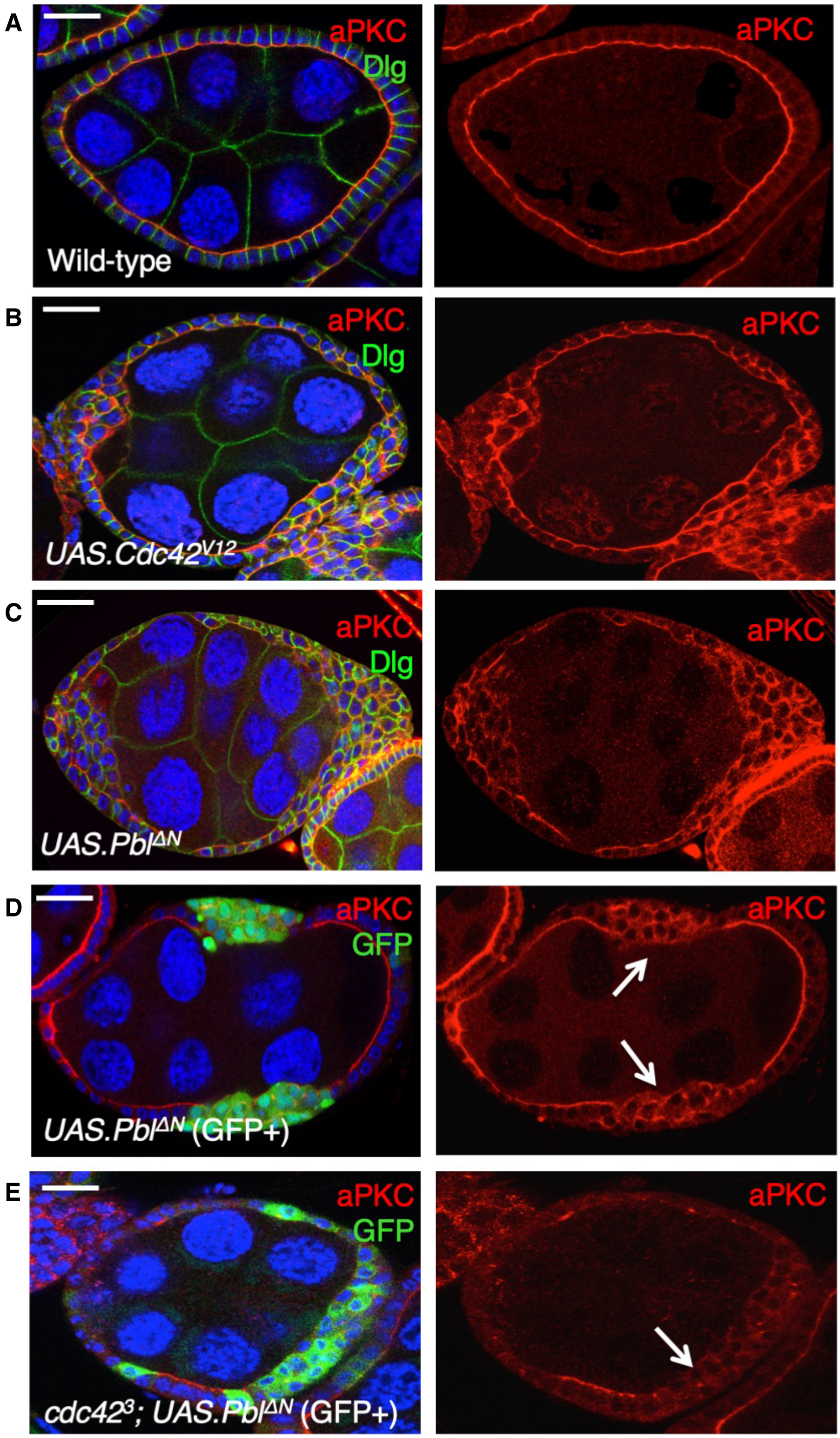

Figure EV5. Overexpression of ECT2/Pbl leads to Cdc42-dependent ectopic spreading of aPKC and cell rounding in the *Drosophila* follicle cell epithelium.

A Wild-type stage 7 egg chamber showing normal localisation of aPKC and Dlg in the follicle cell epithelium. Scale bar ~6 µm. $n > 4$ independent biological replicates.

B *tj.Gal4*-driven *UAS.Cdc42*^{V12} causes ectopic spreading of aPKC in most cells. Scale bar ~6 µm. $n > 3$ independent biological replicates.

C *tj.Gal4*-driven *UAS.Pbl-deltaN* causes ectopic spreading of aPKC in most cells. Scale bar ~6 µm. $n > 6$ independent biological replicates.

D MARCM clonal induction of *UAS.Pbl-deltaN* and *UAS.GFP* causes ectopic spreading of aPKC in most GFP-positive cells. Scale bar ~6 µm. $n > 5$ independent biological replicates. Arrows point to GFP^{+} clones.

E MARCM induction of *UAS.Pbl-deltaN* and *UAS.GFP* in *cdc42*^{3} mutant clones causes loss of aPKC in most GFP-positive cells. This result indicates that overexpressed Pbl acts upstream of Cdc42 to control aPKC localisation. Scale bar ~6 µm. $n > 3$ independent biological replicates. Arrows point to GFP^{+} clones.

© 2020 The Authors

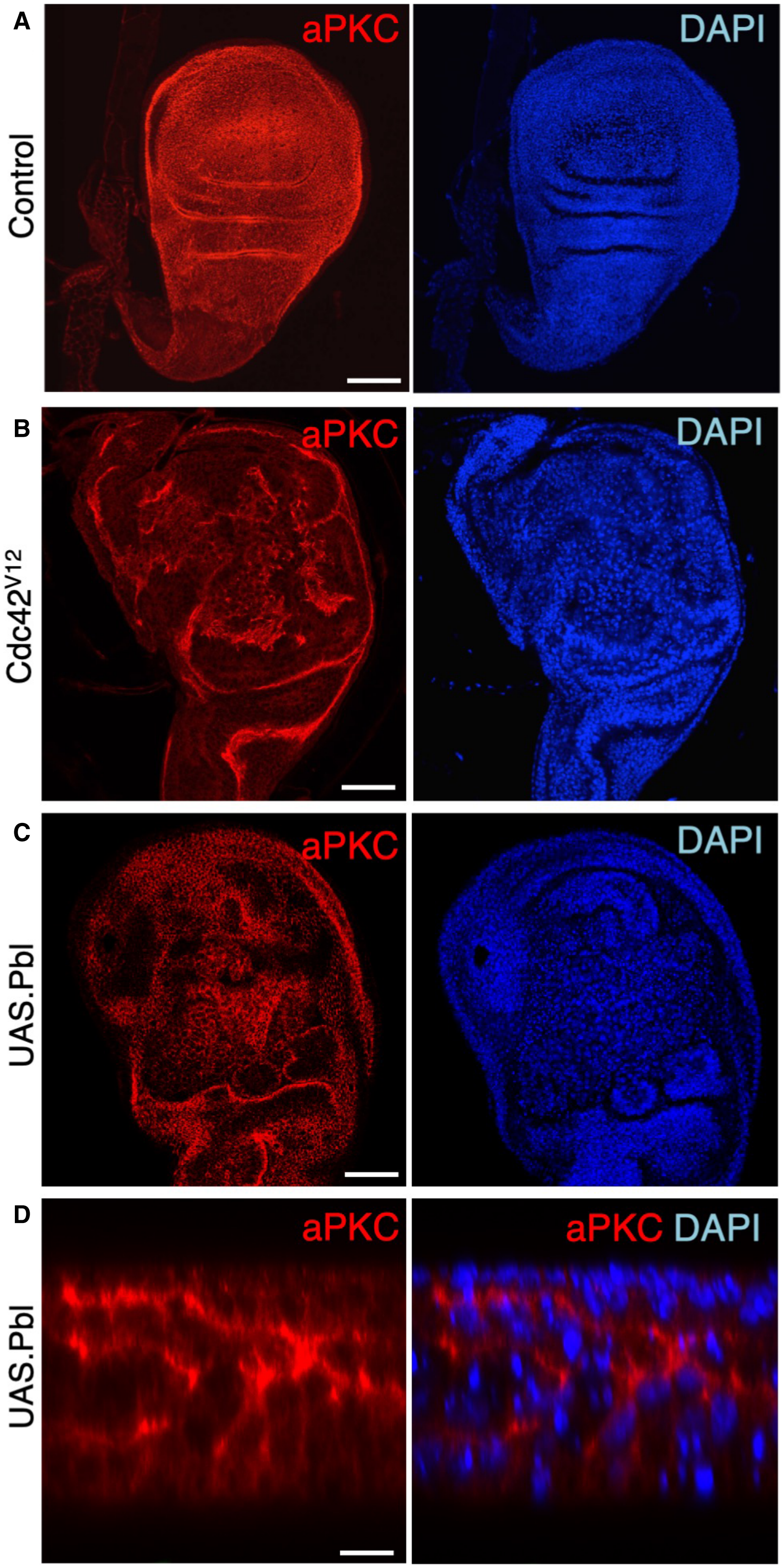

Figure EV6. Overexpression of ECT2/Pbl in the wing imaginal disc leads to ectopic spreading of aPKC and cell rounding in the *Drosophila* wing disc.

- A Control third instar wing disc stained for aPKC and nuclei (DAPI). Scale bar ~20 μm. *n* > 3 independent biological replicates.
- B *MS1096.Gal4*-driven *UAS.Cdc42*^{V12} causes ectopic spreading of aPKC and abnormal tissue morphology. Scale bar ~20 μm. *n* > 6 independent biological replicates.
- C *MS1096.Gal4*-driven *UAS.Pbl* causes ectopic spreading of aPKC and abnormal tissue morphology. Scale bar ~20 μm. *n* > 8 independent biological replicates.
- D High-magnification cross-section of *MS1096.Gal4*-driven *UAS.Pbl* causing ectopic spreading of aPKC and abnormal tissue morphology. The phenotypes of Cdc42 and Pbl gain of function are similar in the wing disc. Scale bar ~2 μm. *n* > 4 independent biological replicates.

 © 2020 The Authors
